# Functional characterization of *Rorippa indica* defensin and its efficacy against *Lipaphis erysimi*

**DOI:** 10.1186/s40064-016-2144-2

**Published:** 2016-04-23

**Authors:** Poulami Sarkar, Jagannath Jana, Subhrangshu Chatterjee, Samir Ranjan Sikdar

**Affiliations:** Division of Plant Biology, Centenary Campus, Bose Institute, Kolkata, 700054 India; Department of Biophysics, Centenary Campus, Bose Institute, Kolkata, 700054 India

**Keywords:** Aphid tolerance, *Brassica juncea* defensin, Mustard aphid, Insect pest management, *Rorippa indica* defensin

## Abstract

*Rorippa indica*, a wild crucifer, has been previously reported as the first identified plant in the germplasm of Brassicaceae known to be tolerant towards the mustard aphid *Lipaphis erysimi* Kaltenbach. We herein report the full-length cloning, expression, purification and characterization of a novel *R. indica* defensin (RiD) and its efficacy against *L*. *erysimi*. Structural analysis through homology modeling of RiD showed longer α-helix and 3rd β-sheet as compared to *Brassica juncea* defensin (BjD). Recombinant RiD and BjD was purified for studying its efficacy against *L. erysimi*. In the artificial diet based insect bioassay, the LC_50_ value of RiD against *L. erysimi* was found to be 9.099 ± 0.621 µg/mL which is far lower than that of BjD (43.51 ± 0.526 µg/mL). This indicates the possibility of RiD having different interacting partner and having better efficacy against *L. erysimi* over BjD. In the transient localization studies, RiD signal peptide directed the RiD: yellow fluorescent protein (YFP) fusion protein to the apoplastic regions which indicates that it might play a very important role in inhibiting nutrient uptake by aphids which follow mainly extracellular route to pierce through the cells. Hence, the present study has a significant implication for the future pest management program of *B. juncea* through the development of aphid tolerant transgenic plants.

**Electronic supplementary material:**

The online version of this article (doi:10.1186/s40064-016-2144-2) contains supplementary material, which is available to authorized users.

## Background

*Brassica juncea* (Indian mustard) is one of the most important crops cultivated in India. More than three dozen insect pests are found to be associated with mustard crops in India, of which mustard aphid (*Lipaphis erysimi* Kalt.) is the most dreaded. These pests are able to reduce the yield up to 96 % (Bakhetia and Sekhon 1989; Singh et al. 1982; Dutta et al. 2005a) and oil content by 15–32 % (Kanrar et al. 2002; Verma and Singh 1987) even after good agricultural practices. Besides causing direct feeding injury these aphids vector many plant viruses (e.g., Turnip mosaic virus and Cauliflower mosaic virus) resulting in further yield loss (Moran and Thompson 2001; Moran et al. 2002) which accounts for approximately 40,006 million of Indian rupees annually (Kular and Kumar 2011). Moreover, the use of unsustainable chemical pesticides by the farmers has increased which are hazardous to human health as well as to the ecosystem. Earlier studies in developing resistant/tolerant *B. juncea* lines against this particular aphid include production of hybrid lines of *B. juncea* with *Brassica fruticulosa* (Atri et al. 2012) and *Brassica campestris* (Goomber and Labana 1983), selection of vigorous lines from the wild type (Abraham and Bhatia 1994), as well as introducing potent insecticidal agents like proteases (Rahbe et al. 2003) and lectins (Dutta et al. 2005a; Kanrar et al. 2002; Mondal et al. 2006) derived from distant plant families. There is no report of resistant/tolerant gene available in the wild germplasm till date. Moreover, in due course of time, a particular insecticidal agent may become ineffective against the pest due to acquired resistance or behavioural reorientation (Chen 2008), entailing the discovery of a new insecticidal agent. Previous study by Bandopadhyay et al. (2013) gave a first molecular insight into crucifer defense response against mustard aphid *L. erysimi*. On wild germplasm screening, *Rorippa indica* (L.) Hiern, a wild crucifer and an occasional shade loving weed was noted to be tolerant against mustard aphid. *R. indica* is found in the Indian subcontinent and Asia (Mandal and Sikdar 2003) which remains in rosette form throughout the winter but subsequently bolts out and grows into highly branched bush throughout the summer. Transcriptomic analysis of *R. indica* in response to *L. erysimi* attack was studied by cDNA AFLP analysis in which thirty unique expressed sequence tags (ESTs) were seen to be differentially regulated (Bandopadhyay et al. 2013). One of the major identified ESTs was found to be homologous to PDF1.2c (plant defensin) of Arabidopsis (GenBank Accession—JK034054) which has been named as *Rorippa* defensin (*RiD*) for the present study.

Plant defensins are basic, cysteine-rich peptides of about 5–8 kDa (45–54 amino acids) and belong to the γ-thionin family (Carvalho and Gomes 2009, 2011; Lacerda et al. 2014). Two classes of defensins have been predicted according to the structure and sequences (Lay and Anderson 2005; Lay et al. 2014). Class-I defensin comprises of an endoplasmic reticulum (ER) signal sequence and a mature domain. This class of proteins enter the secretory pathway and do not undergo post-translational modification or subcellular targeting. Class-II defensin contains a C-terminal pro-domain of about 33 amino acids (Lay et al. 2003; Vriens et al. 2014). Many defensins show antifungal (Terras et al. 1992, 1993), antibacterial (Koike et al. 2002; Zhang and Lewis 1997) and insecticidal (Chen et al. 2002) activity. Plant defensins also act as proteinase inhibitors (Melo et al. 2002), protein synthesis inhibitor (Harrison et al. 1997), α-amylase inhibitor (Bloch and Richardson 1991), and sodium channel inhibitor (Kushmerick et al. 1998) and can inhibit insect feeding by destroying midgut activity (Carvalho and Gomes 2009; Lin et al. 2007; Pelegrini and Franco 2005; Pelegrini et al. 2008; Santos et al. 2010). Insecticidal and α-amylase inhibitory activities of defensins are also reported in *Vigna radiata* (Liu et al. 2006) as well as in *Sorghum bicolor* (Bloch and Richardson 1991). Cowpea seed defensin was seen to inhibit α-amylase from the weevils—*Callosobruchus maculatus* and *Zabrotes subfasciatus* (Santos et al. 2010). BrD1, a defensin gene from *Brassica rapa*, provides resistance from brown planthopper (*Nilaparvata lugens*) in transgenic rice (Choi et al. 2009).

In the aforementioned background, the present study was conducted to characterize and study the efficacy of RiD against *L. erysimi*, over *Brassica juncea* defensin (BjD). Homology modelling was done to study the differences between the model structures of RiD and BjD. The RiD and BjD coding sequence were cloned, expressed and purified using prokaryotic expression system. For further characterization, transient localization of RiD was studied and finally the insecticidal potential of expressed RiD as well as that of BjD was checked through artificial diet based insect bioassay against *L. erysimi*.

## Results

### Aphid infestation study

**Fig. 1:**
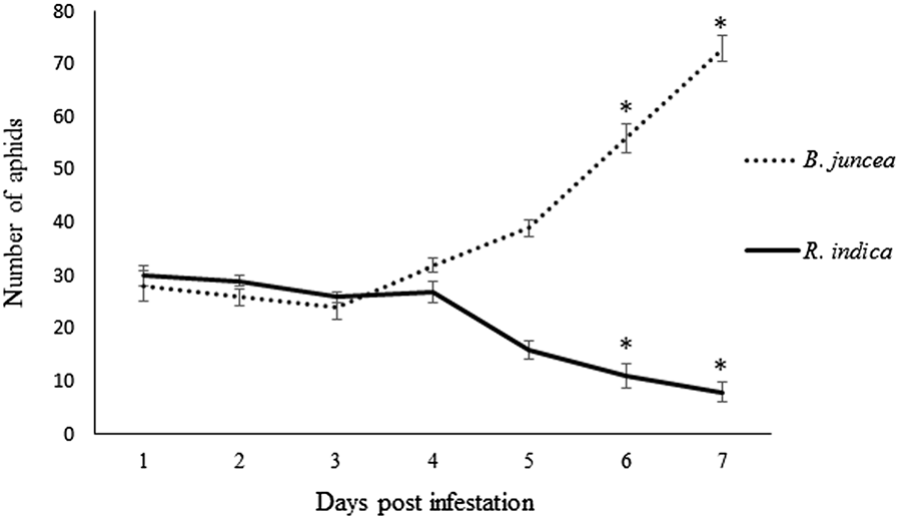
Aphid population on *R. indica* and *B. juncea.* The total number of live aphids were recorded over 7 days post infestation at an interval of 24 h. Maximum colonization was observed in case of *B. juncea.* Whereas a decrease in population of aphids was seen in *R. indica* after 4 dpi. *Bars* represent standard error (SE) where number of independent experiments (n) = 3. The significant changes (P ≤ 0.05), marked by *asterisk* were analyzed by Student’s t test

Time course aphid population study was carried out for up to 7 days. After infesting each plant with 30 aphids, a settling time of 2 h was allowed. Following this, the number of viable wingless aphids per plant was counted at each day post infestation. A surge in the population of nymphs and aptera was noted within 4 days post infestation (dpi) (Fig. 1). After 4 dpi, population in *R. indica* started to decline sharply with the appearance of the winged (alate) form of the aphids. Among all the sets of infested and non-infested plants as shown in Fig. 1, *R. indica* was seen to have lower aphid population with increasing days post infestation than *B. juncea*. The challenged *R. indica* plants were found to show normal development like the non-infested control plants (Additional file 1: Fig. S1).

### Time course semi-quantitative RT-PCR and real-time PCR analysis

**Fig. 2:**
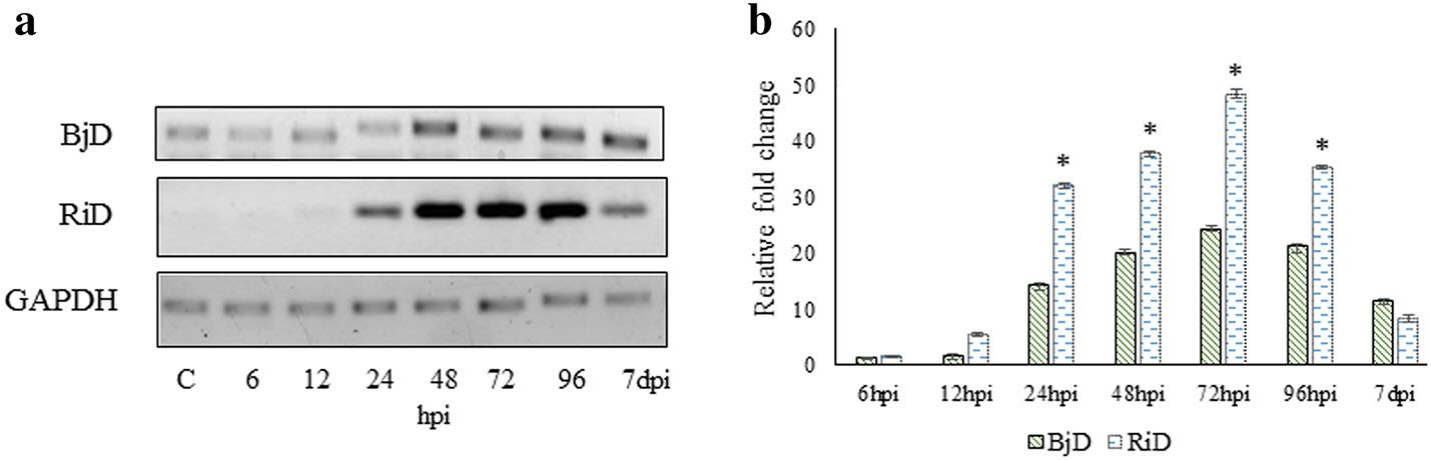
**a** Semi quantitative PCR analysis of PDF1.2c homolog in *R. indica* (RiD) and *B. juncea* (BjD) at different time points. **b** Real time expression profile of PDF1.2c homolog in *R. indica* and *B. juncea* upon aphid infestation. *Bars* represent standard error (SE) of three biological replicates (n). The significant changes (P ≤ 0.01) were marked by *asterisk* (analyzed by Student’s t test)

Semi-quantitative expression of PDF1.2c homolog in *R. indica* as well as in *B. juncea* was studied using the primers from the EST as previously described (Additional file 4: Table S1). The RT-PCR profile is presented in Fig. 2a. A 1.5-fold up-regulation was observed in *R. indica* early at 6 h as compared to 1.2-folds in *B. juncea* and the expression culminated to 48.2-folds in *R. indica* by 72 h post infection (hpi) through 9.45 and 9.67-fold at 24 and 48 h respectively (Fig. 2b). Whereas, PDF1.2c homolog expression in *B. juncea* reached a peak not more than 24.25-folds by 72 hpi and declined to 11.5-folds by 7 dpi. The expression level of PDF1.2c homolog in *R. indica* drops to 8.2-folds by 7 dpi (Fig. 2b).

### Determination of the full-length coding sequence of *RiD* and *BjD* and in silico analysis

**Fig. 3:**
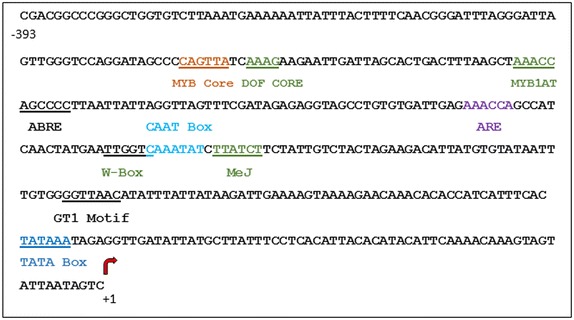
In silico promoter analysis showing important cis acting elements. A 393 bp region was analyzed for upstream elements (ARE: cis-acting regulatory element essential for the anaerobic induction, ABRE: abscisic acid response element, DOF Core: DNA-binding domain with one finger transcription factors, GT1 Motif: light responsive element, I-Box: part of a light responsive element, MeJ: methyl jasmonate inducing region, MYB Core: MYB transcription factor binding region, MYB1AT: dehydration responsive element, TATA Box: core promoter element around −30 of transcription start, W-Box: binding sites for WRKY transcription factors)

5′ and 3′ RACE was carried out to identify the full-length coding sequence of *R. indica* defensin (243 bp) and *B. juncea* defensin (243 bp). A single intron in the *RiD* gene and a short region of 393 bp upstream element containing TATA box and other cis-acting elements were also identified (Fig. 3) using the protocols of genome walk. The coding sequences and upstream elements of RiD and BjD were submitted to GenBank with accession numbers KP893333 and KU513489 respectively.

**Fig. 4:**
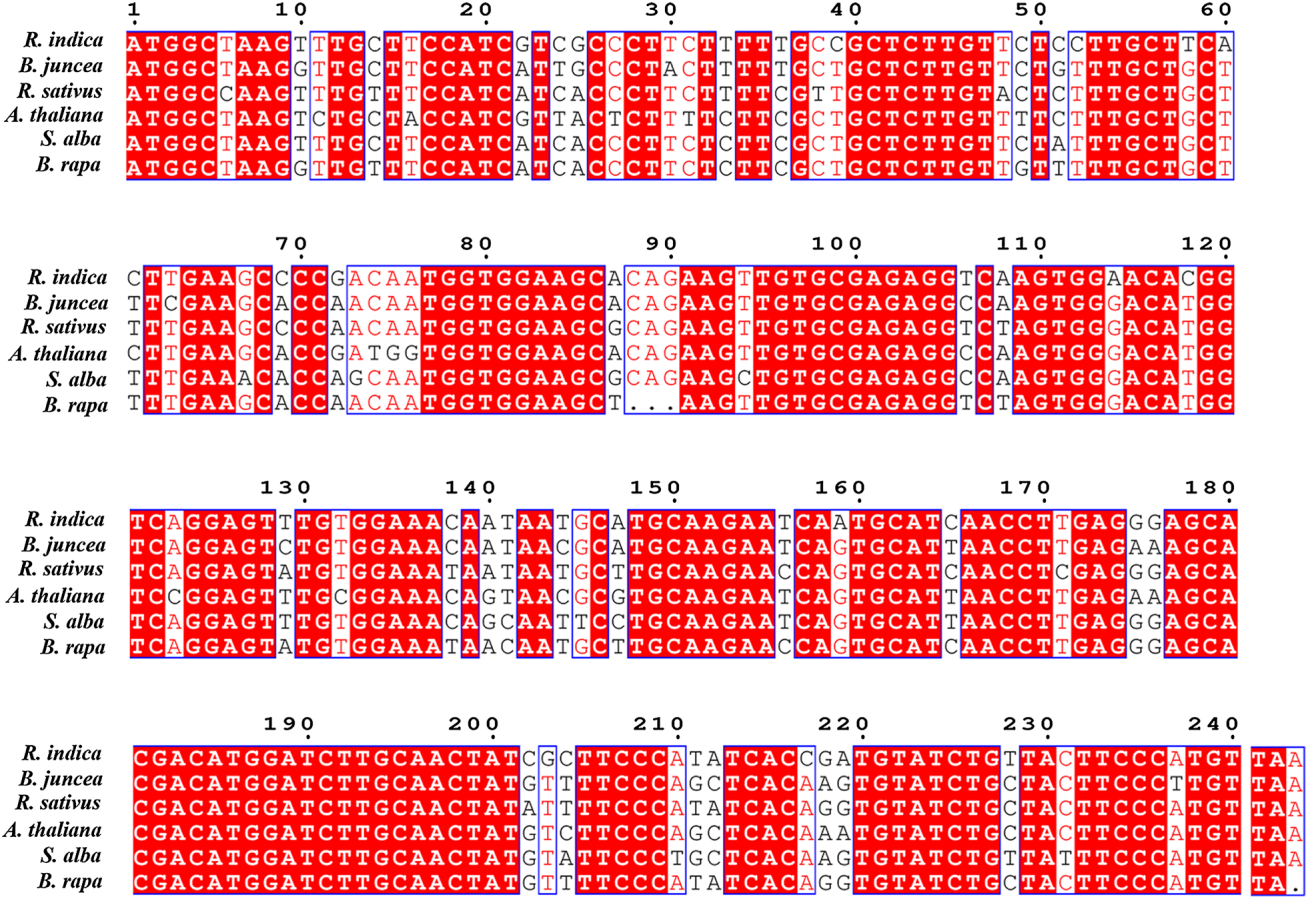
Sequence alignment of *RiD* (GenBank accession—KP893333) with that of other plant defensins of Brassicaceae, viz. *B. juncea* (GenBank accession—KU513489), *Raphanus sativus* (GenBank accession—U18557), Arabidopsis (GenBank accession—NM106233.3), *Sinapis alba* (GenBank accession—AY998243) and *Brassica rapa* (GenBank accession—XM009106448)

The sequence of *RiD* was aligned with other plant defensins of the same family—Brassicaceae along with *BjD*. *RiD* showed 87, 88, 85, 86 and 86 % identity with the defensins from *B. juncea*, *Raphanus sativus* (GenBank accession—U18557), Arabidopsis (GenBank accession—NM106233.3), *Sinapis alba* (GenBank accession—AY998243) and *Brassica rapa* (GenBank accession—XM009106448) respectively at nucleotide level (Fig. 4).

### Homology modelling and structure determination

**Fig. 5:**
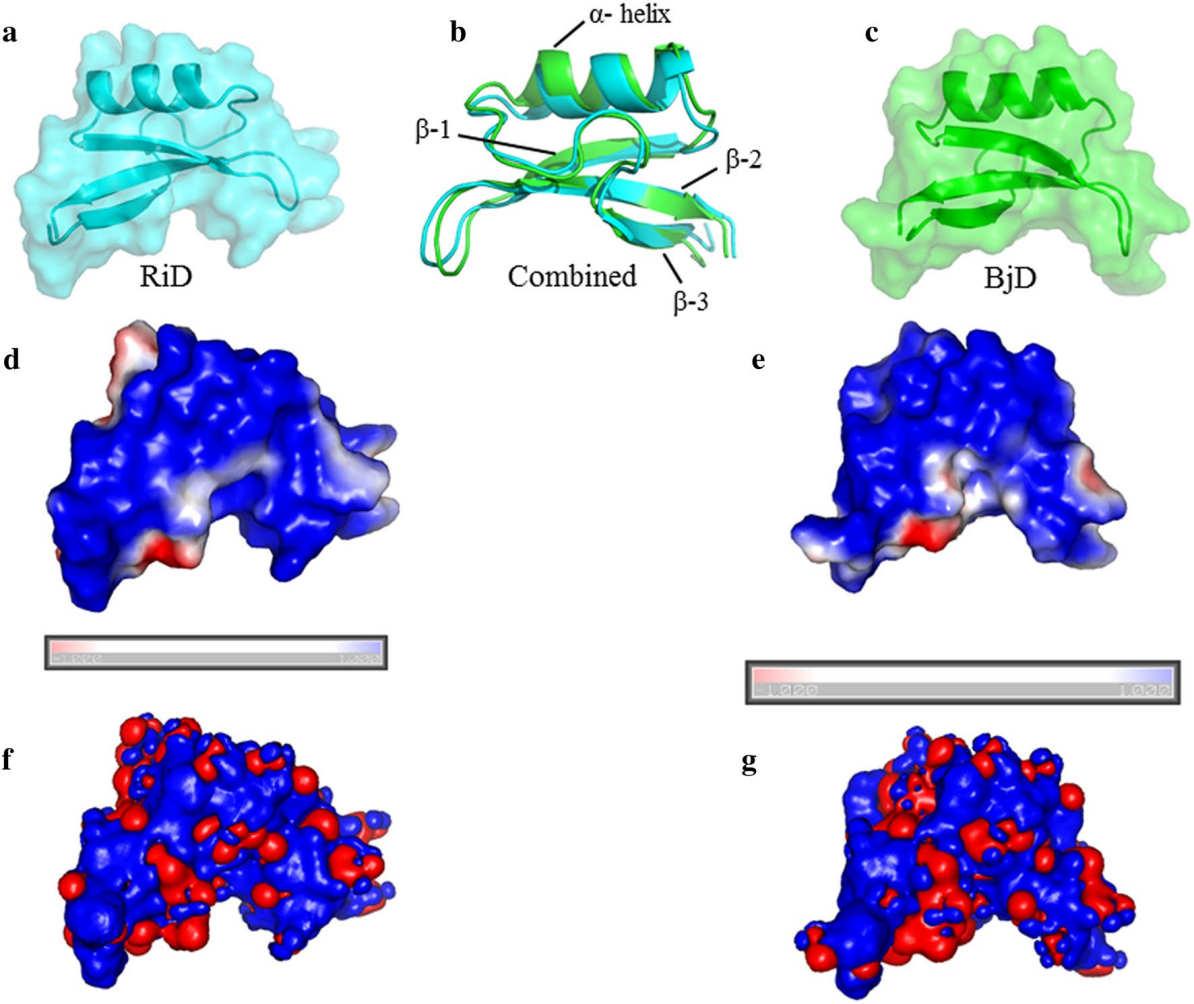
Homology model structure of RiD (**a**) and BjD (**c**) showing one α-helix and three β-sheet region. Superimposed homology model structure of RiD (*cyan*) and BjD (*green*) (**b**). *Color* by potential of solvent accessible surface of RiD (**d**) and BjD (**e**) have been calculated using APBS. The *bars* indicates color by potential [positive (*blue*) and negative (*red*)]. Electrostatic iso-surfaces of RiD (**f**) and BiD (**g**) are also shown

RiD and BjD both showed 98 % sequence identity at amino acid level with *R. sativus* antifungal protein 1 (PDB ID: 1AYJ). Homology modelling clearly shows that despite having 86 % similarity at the amino acid level, the α-helix and 3rd β-sheet of RiD are little longer as compared with BjD (Fig. 5a–c).

### Adaptive Poisson–Boltzmann Solver (APBS) iso-surface calculations

The electrostatic potential and distribution of atomic partial charge of biomacromolecule in connection with dielectric constant could be explained by APBS. The Adaptive Poisson–Boltzmann Solver (APBS) was used to calculate the electrostatic surface of the proteins. For both RiD and BjD, the outer surfaces of the proteins were seen to have more positive charge (Fig. 5d–g).

### Protein expression and purification

**Fig. 6:**
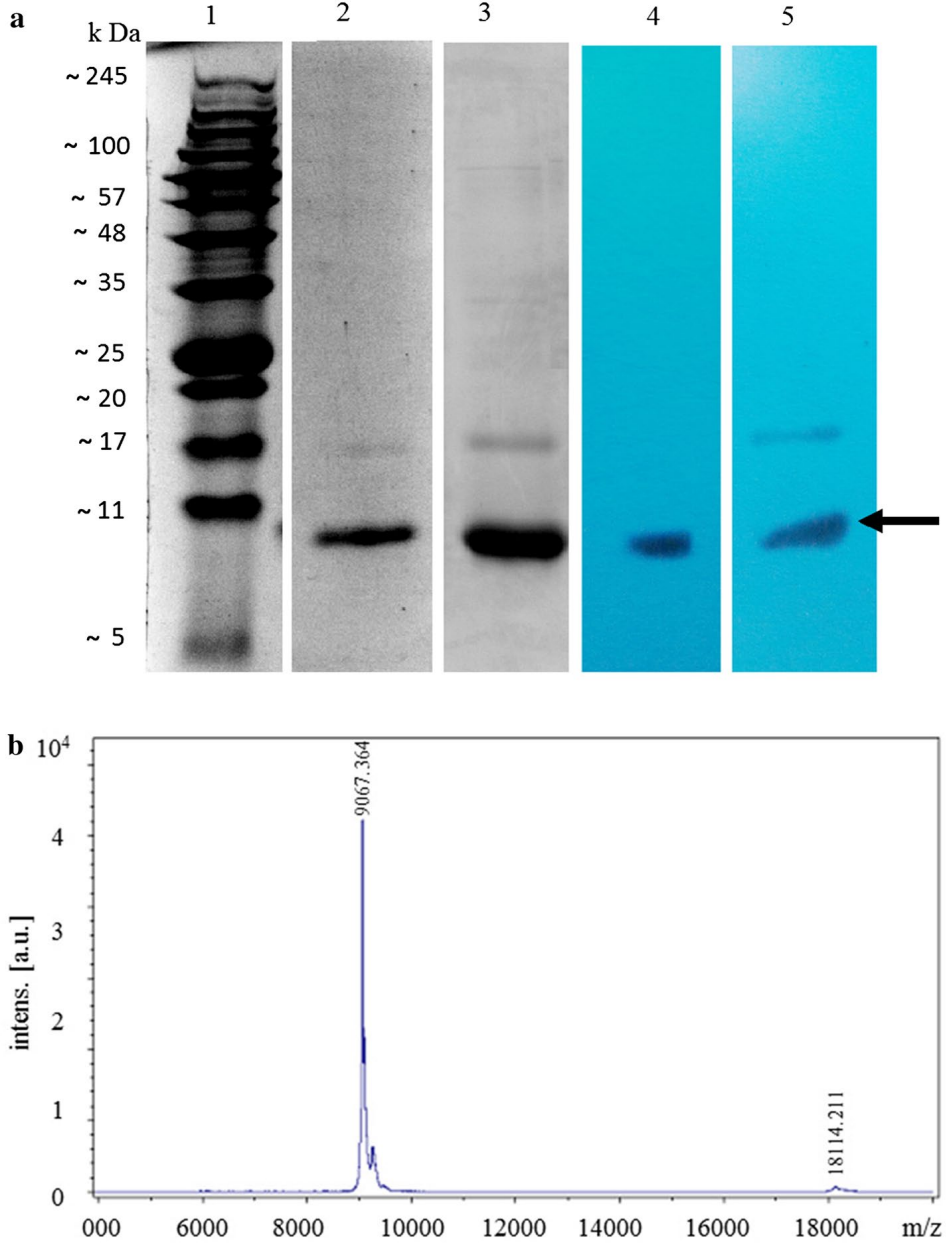
Purification of RiD and BjD. **a** *Lane 1* Protein marker, *Lane 2* purified RiD, *Lane 3* purified BjD, *Lane 4* Western blot using anti-His antibody (1:5000) against purified RiD, *Lane 5* Western blot using anti-His antibody (1:5000) against purified BjD. *Arrow* indicates purified proteins. **b** Determination of RiD molecular mass by MALDI-TOF/TOF–MS spectrometry. A *sharp peak* confirms the quality of purification of RiD

The recombinant pET28a+ vectors harbouring the coding sequence of *RiD* and *BjD* were transformed into Rosetta (DE3) pLysS cells individually. The expression constructs produced a 6His-RiD and 6His-BjD fusion protein respectively, which were purified by Ni–NTA column. The maximum expression of RiD and BjD was obtained at 1 mM IPTG at 20° C after 16 h of incubation. As shown in Fig. 6a, lane 2 and lane 3, bands of approximately 9 kDa of both RiD and BjD were eluted from the induced clones in 12 % SDS-PAGE gel. The purified proteins were further validated by western blot analysis using anti-His antibody (Fig. 6a, lanes 4 and 5).

### Mass spectrometric analysis of purified RiD

A MALDI-TOF/TOF–MS analysis was performed to verify the molecular mass of the purified RiD protein. MS analysis yielded a mass of 9067.364 Da for the purified peptide (Fig. 6b). The molecular mass of RiD is ~6 kDa and the size of the tag (6His) associated with the defensin is ~3 kDa. Thus, the recombinant protein obtained was of ~9 kDa. The MALDI-TOF/TOF–MS spectrum was generated from in-solution trypsin digestion (Additional file 2: Fig. S2). Furthermore, analyses by the MASCOT search program in the NCBI database suggested that the obtained sequence is highly similar with *R. indica* defensin, and other defensin-like proteins. The observed monoisotopic mass of tryptically digested peptides obtained by MALDI-TOF/TOF–MS and their position with respect to matched protein sequence are presented in Table 1. The matched peptides of RiD covered 33 % of defensin (*R. indica*, gi694199016) with score 47 and 48 % with defensin-like protein 1 (*Camelina sativa*, gi727556613) with score 50.

**Table 1:** Summary of matched peptides of RiD analyzed by MALDI-TOF/TOF–MS

Protein name/accession number	Theoretical pI	Score	Peptide mass (Da)	Position	Matched peptides
Defensin (*Rorippa indica*, 694199016)	8.71	47	507.3724	36–40	R. SSGTR. S
			719.4432	69–73	R. FPYHR. C
			1174.6655	52–61	K. NQCINLEGAR.H
			1593.8500	62–73	R.HGSCNYRFPYHR.C
Defensin-like protein 1 (*Camelina sativa*, 727556613)	8.42	50	719.4432	67–71	R.FPYHR.C
			1174.6655	50–59	K.NQCINLEGAR.H
			1593.8500	60–71	R.HGSCNYRFPYHR.C
			1698.8252	34–49	R.SSGTWSGVCGNNNACK.N
Defensin-like protein 3 (*Camelina sativa*, 727556613)	8.42	49	719.4432	68–72	R.FPYHR.C
			1174.6655	51–60	K.NQCINLEGAR.H
			1593.8500	61–72	R.HGSCNYRFPYHR.C
			1698.8252	35–50	R. SSGTWSGVCGNNNACK.N

### Localization of RiD

**Fig. 7:**
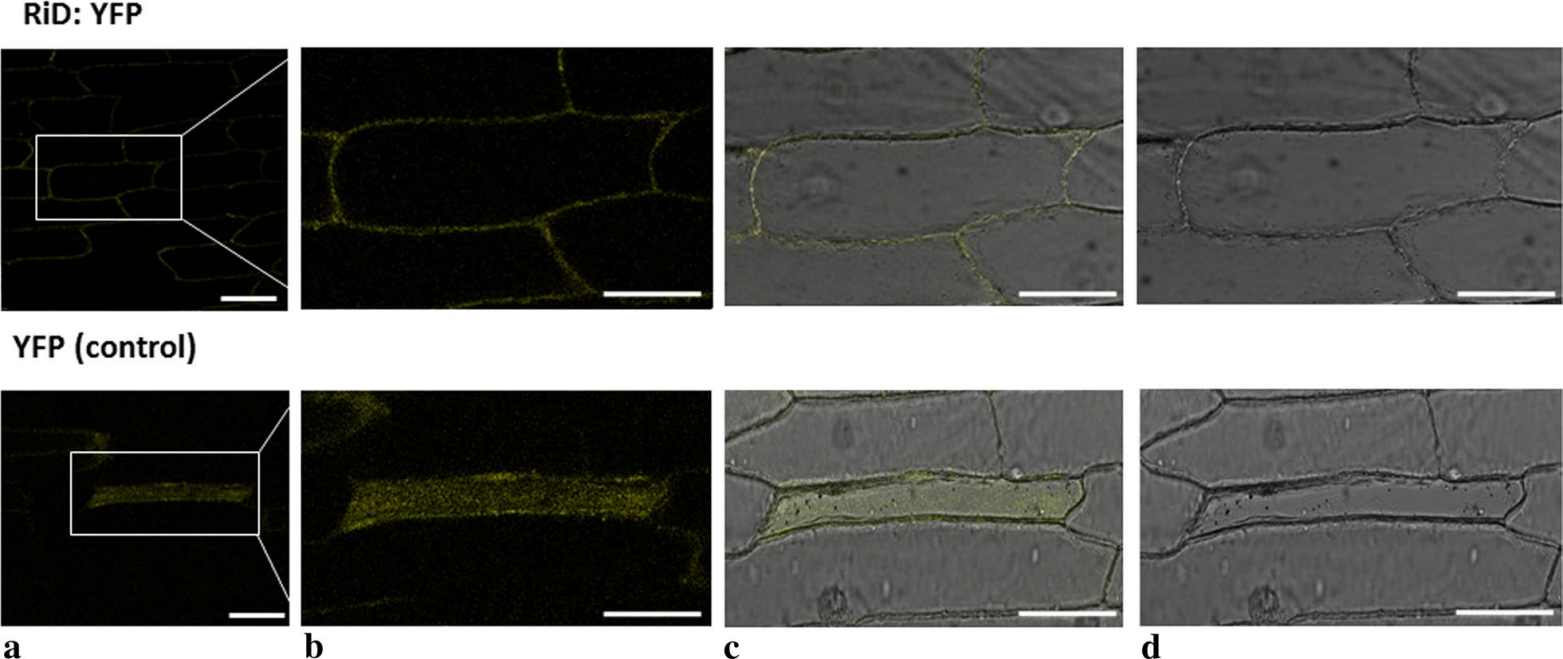
Localization studies of RiD:YFP and YFP only (as control) in onion epidermal cells. Confocal laser sections show YFP fluorescence in onion epidermal cells expressing RiD:YFP in the apoplastic regions and diffused fluorescence in the cells expressing only YFP. **a** Fluorescence, **b** magnified fluorescence, **c** merged, **d** bright field. (Magnification: ×20). *Bar* 75 µm

All class-I defensin have a predicted N-terminal signal peptide supposed to translocate the defensin into the lumen of the endoplasmic reticulum (ER) and thus to the secretory pathway. A SignalP analysis (Petersen et al. 2011) of the *R. indica* defensin showed that RiD also has a putative N-terminal secretory signal peptide (Additional file 3: Fig. S3a). In order to determine the function of this putative defensin, chimeric proteins were constructed with yellow fluorescent protein (YFP) fused to the C-terminus of the full-length RiD (RiD: YFP) (Additional file 3: Fig. S3b) and only YFP lacking RiD was used as a control. RiD: YFP labelling was observed in the apoplastic regions of the onion epidermal while fluorescence from control YFP was seen to be diffused in the cytoplasm (Fig. 7).

### Insect bioassay on artificial diet

**Fig. 8:**
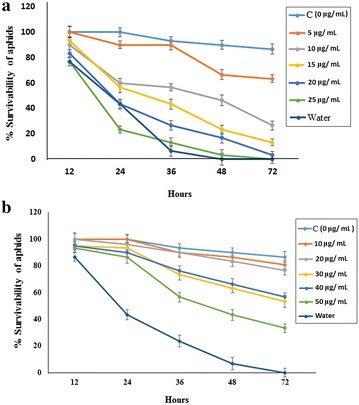
Insect bioassay in artificial diet supplemented with RiD and BjD on second instar nymphs of *L. erysimi*. Insect survivability graph at different concentrations of **a** RiD (0, 5, 10, 15, 20, 25 µg/mL) and **b** BjD (0, 10, 20, 30, 40, 50 µg/mL) were recorded over 72 h

Mortality of *L. erysimi* was studied using artificial liquid diet supplemented with different concentrations of RiD (Fig. 8a) and BjD (Fig. 8b). Liquid diet with no RiD and no BjD was used as control. The total mortality (*P*′) was calculated from the Abbot’s formula (Abbott 1925), *P*′ = *C* + *P* (1 − *C*), considering a population (*P*) which would have survived in the absence of the insecticidal agent used in the experiment and where C is natural mortality without the agent. LC_50_ value of RiD and BjD against *L. erysimi* were calculated accordingly using the statistical Probit analysis. LC_50_ value of RiD against *L. erysimi* was found to be 9.099 ± 0.621 µg/mL, whereas of BjD was found to be 43.51 ± 0.526 µg/mL (Table 2).

**Table 2:** LC_50_ value of purified RiD and BjD against *Lipaphis erysimi* in artificial diet based bioassay

Protein used	LC_50_ value (µg/mL)	Lower fiducial limit	Upper fiducial limit	SE of slope	Regression equation Y	χ^2^ value	df
RiD	9.099	13.726	3.274	0.621	7.921 + (−3.046)x	3.816	3
BjD	43.51	48.321	37.624	0.526	1.77 + 1.60 x	1.210	3

## Discussion

Plant defensins are known to have a diverse range of functions. They are one of the most important components of the innate immune system that have been conserved during evolution. Among the limited insect resistance genes like *Bacillus thuringiensis* (Bt) genes (Lawo et al. 2009; Porcar et al. 2009), proteases (Rahbe and Febvay 1993) and lectins (Bandopadhyay et al. 2001; Kanrar et al. 2002; Banerjee et al. 2004; Dutta et al. 2005a, b; Mondal et al. 2006; Das et al. 2013), till date only lectins have been studied to be effective against *L. erysimi* (Dutta et al. 2005b; Kanrar et al. 2002; Mondal et al. 2006; Roy et al. 2014). Defensins with α-amylase inhibitory activity are potential candidates which can possibly provide resistance against insect feeding (Pelegrini et al. 2008) and can be a promising tool in transgenic development strategies in this present scenario.

In the present study, a novel defensin gene—*RiD* has been isolated and characterized from *R. indica*, previously noted to be aphid tolerant by Bandopadhyay et al. (2013). RiD is seen to be highly upregulated when infested upon by the mustard aphid—*L. erysimi* (Bandopadhyay et al. 2013). Here, RiD and defensin from *B. juncea* (BjD) has been successfully cloned, expressed, purified and characterized. Analysis of RiD and BjD through homology modelling showed structural difference through the presence of longer α-helix and 3rd β-sheet in the former one, despite having significant similarity in nucleotide level. Plant defensins are reported to have a broad spectrum of biological activities (Lacerda et al. 2014). Reports of VrD1 from *Vigna radiata* is shown to inhibit insect α-amylase whereas VrD2 from the same plant does not have any insecticidal activity nor α-amylase inhibitory activity (Lin et al. 2007). There is also a family of defensins isolated from *Vigna unguiculata*, in which different homologous forms may act as antifungal, antibacterial, and enzyme inhibitors (Franco 2011). Thus, RiD being very identical to BjD might have a different mode of action and efficacy against *L. erysimi*.

Transient expression of RiD fused with YFP showed that RiD is secreted into the apoplast and not retained in the intracellular compartments. Secretion into the apoplast is consistent with the expected function of the signal peptide of all Class-I defensins, allowing a post-translational translocation into the lumen of ER. This is in accordance with other plant defensins (Kaur et al. 2012; Kragh et al. 1995) and contradictory to AhPDF1.1 of Arabidopsis, where it is retained in the vacuoles (Oomen et al. 2011). As aphid stylets follow mainly extracellular pathway through the apoplast to obtain nutrition (Jaouannet et al. 2014; Will and Vilcinskas 2015) RiD might play a very important role in inhibiting sap sucking by aphids.

To determine the insecticidal efficacy of RiD against *L. erysimi*, it is essential to calculate the LC_50_ value of the protein against the target insect. Hence, artificial diet based bioassay was conducted and it was observed that RiD induced mortality in *L. erysimi* with LC_50_ value of 9.099 ± 0.621 µg/mL. Similar bioassay conducted using BjD yielded LC_50_ value of 43.51 ± 0.526 µg/mL, indicating that BjD is not at all effective against this aphid. The LC_50_ value of RiD was also observed to be lower than that of other insecticidal agents such as *Amorphophallus paeonifolius* tuber agglutinin (AMTL) with LC_50_ value of 13.47 ± 0.23 µg/mL (Mondal et al. 2012), *Allium sativum* leaf agglutinin (ASAL) with LC_50_ value 20.7 ± 0.21 µg/mL (Banerjee et al. 2011), *Colocasia esculenta* tuber agglutinin (CEA) with LC_50_ value of 11.87 ± 0.229 µg/mL (Roy et al. 2014) and proteases with LC_50_ value of 22 µg/mL (Harrison and Bonning 2010). This indicates the suitability of RiD as an insecticidal agent over BjD and other previously reported agents against *L. erysimi*. Further studies in identifying any interacting partners from *L. erysimi* might provide a better insight into the mechanism of RiD. To the best of our knowledge, this is the first report of characterization of a putative mustard aphid tolerant gene from the Brassicaceae germplasm.

## Conclusion

In the present study, RiD and BjD were isolated from *R. indica* and *B. juncea* respectively. Despite the high level of homology on sequence level with BjD, RiD has been seen to differ in its structural and surface properties as well as its efficacy against *L. erysimi*, suggesting that RiD could be a potent insecticidal agent. Hence, the present study might be promising for the development of aphid tolerance in *B. juncea* through the generation of transgenics.

## Methods

### Experimental set up

*Rorippa indica* and *B. juncea* plants of 60 days old were grown under laboratory conditions under 16 h photoperiodic light (3000 lux), temperature of 25 ± 2 °C and 75 % relative humidity. Mustard aphids (*L. erysimi*) were collected from the Madhyamgram Experimental Farm, Bose Institute, India. Thirty aphids of third-instar stage were forcedly infested upon each plant (under laboratory conditions previously mentioned), which were used for further experiments. Three plants with 30 aphids each were used as replicates.

### Aphid population

The total aphid population was studied in both *R. indica* and *B. juncea* at an interval of every 24 h for 7 days, with three plants for each time point. Student’s t test at a significance level of P ≤ 0.05 was carried out to determine the significance of the infestation noted over the time course.

### RNA isolation, cDNA preparation and rapid amplification of cDNA ends

Total RNA was isolated from the aerial parts (leaves and stem) of all the infested plants at different time points as well as the non-infested control plants using Trizol reagent (Invitrogen, CA, USA). First strand cDNA was synthesized using iScript-cDNA synthesis kit (Bio-Rad, CA, USA). The obtained cDNA was used for semi-quantitative analysis for the expression of PDF1.2c homolog and real-time PCR analysis using iQ SYBR Green Supermix (Bio-Rad Laboratories) in a iQ5 Multicolor Real-Time PCR Detection System (Bio-Rad Laboratories). Primers were designed on the basis of the previously identified EST (GenBank Accession—JK034054) (Bandopadhyay et al. 2013). The reactions were studied at all seven time-points with three different biological replicates. The real-time RT-PCR condition used was as follows: initial denaturation at 95 °C (3 min), followed by 50 cycles (10 s) at 95 °C and 30 s at 55 °C. GAPDH was used as an internal control. The average fold value changes for the different time points were calculated using 2^−∆∆Ct^ method (Livak and Schmittgen 2001). The full-length mRNA sequence was determined using 5′ and 3′ RACE System for rapid amplification of cDNA ends (Invitrogen, CA, USA).

### Genomic DNA isolation and genome walk

Genomic DNA of *R. indica* was isolated using DNeasy Plant maxi kit (Qiagen, Hilden, Germany). Genome walk was carried out according to the protocols of BD genome Walker™ Universal Kit (BD Biosciences Clontech, CA, USA). The primers were designed according to the sequence derived from RACE (Additional file 4: Table S1). PCR product was purified using Qiaquick Gel Extraction kit (Qiagen, Hilden, Germany) and was cloned into pGEMT Easy Vector (Promega, Wisconsin, USA). Nucleotide sequencing of the clones was performed using Applied Biosystems (USA) 3130 *xl* Genetic Analyzer at the sequencing facility of Bose Institute, Kolkata, India. RiDForward and RiDReverse primers were used to clone the full-length gene sequence of *RiD* (Additional file 4: Table S1).

### In silico analysis

The coding sequences of *RiD* and *BjD* derived from RACE were analyzed in TAIR and NCBI resources using BLASTN algorithm (Altschul et al. 1990). All the sequences derived from the Brassicaceae family were aligned using ClustalW (Thompson et al. 1994) and ESPript 3.0 (Robert and Gouet 2014). The intron in *RiD* gene was identified using GENSCAN (Burge and Karlin 1997) as well as aligning the sequence to the full-length cDNA sequence. The sequence was also analyzed using PLACE (Higo et al. 1998) and PlantCARE (Lescot et al. 2002) for identification of upstream elements.

### Homology modelling

The full-length coding sequence of *RiD* and *BjD* were translated using ExPASy translate tool. The primary amino acid sequences thus derived were taken to investigate for a proper template in Protein Data Bank (PDB) to generate 3D coordinates of both RiD and BjD. Both the sequences were searched for a structure using Prime v3.4 module of Schrödinger molecular modelling suite (Jacobson et al. 2004). BLAST homology search was performed against non-redundant protein databank. The structures derived from homology modelling were further energy minimized using Polak-Ribiere conjugate gradient (PRCG) method, where the maximum iteration steps were 2500. The whole method was performed using OPLS_2005 force field (Jorgensen et al. 1996). The structure derived for RiD was also aligned with that of BjD in PyMol to identify the regions of dissimilarity present if any.

### Adaptive Poisson–Boltzmann Solver (APBS) iso-surface calculations

The electrostatic characteristics of the protein were analyzed using APBS (Baker et al. 2001). The AMBER force field was used for the generation of standard all-atom charges. The various computations necessary to calculate the electrostatic properties were performed using a plugin option of PyMOLv1.2r3pre. The electrostatic contour visualizations were collected at the suitable positive and negative iso-surface values, optimized for visualization.

### Protein expression and purification

The full-length coding sequence of *RiD* and *BjD* encoding the mature peptide was cloned into the pET28a+ vector individually (Novagen, WI, USA) using RiDexp-F/RiDexp-R (for RiD) and BjDexp-F/BjDexp-R (for BjD) containing the *Bam*HI (in forward primers, underlined) and *Sac*I (in reverse primers, underlined) sites (Additional file 4: Table S1). The recombinant plasmids were then transformed into the *E. coli* Rosetta (DE3) pLysS cell line (Novagen, WI, USA). The recombinant cells were induced with 1 mM isopropyl-β-d-thiogalactopyranoside (IPTG) and incubated with constant shaking at 20 °C for 16 h. The recombinant cells were pelleted by centrifugation at 5000*g* at 4 °C for 30 min, resuspended in lysis buffer (50 mM NaH_2_PO_4_, 300 mM NaCl, 10 mM imidazole, pH 7.4) and sonicated 30 times for 30 s each. The cell suspension was centrifuged at 10,000*g* for 30 min at 4 °C, and the supernatant was incubated for 2 h in 2 mL of nickel–nitrilotriacetic acid (Ni–NTA) column equilibrated with lysis buffer. The column was washed with wash buffer with 20 mM imidazole, pH 7.4 to remove nonspecific proteins, and finally, the target fusion protein was eluted with elution buffer with 250 mM imidazole, pH 7.4. All purification steps were carried out at 4 °C. The expression and purification of the recombinant proteins were analyzed in 12 % SDS-PAGE stained with Coomassie Brilliant Blue. The expressions of the proteins were further confirmed by western blot analysis using anti-His antibody (1: 5000) (Biobharati, Kolkata, India).

### MALDI-TOF/TOF–mass spectrometric analysis

Protein structures may lead to anomalously fast migration in SDS-PAGE (Jong et al. 1978, Rath et al. 2009). To determine the exact molecular mass, purified RiD was analyzed on a matrix-assisted laser desorption/ionization time-of-flight Autoflex III mass spectrometer (Bruker Daltonics, Germany). Samples were prepared by mixing equal volumes of 0.1 % trifluoroacetic acid (TFA), acetonitrile (1:1) and the protein solution. A 2 µL portion of the above sample was mixed with 2 µL of freshly prepared α-cyano-4-hydroxycinnamic acid (HCCA) matrix in 50 % acetonitrile and 1 % TFA (1:1), and 2 µL was spotted on the target Anchor Chip MALDI plate (Bruker Daltonics, Germany). The protein was also digested with trypsin (Promega, Madison, USA) according to the in solution digestion protocol of Mann (2006). The spectra obtained were analyzed with Flex Analysis Software (version 2.4, Bruker Daltonics, Germany) for deletion of matrix peaks and tryptic autolysis peaks. Processed spectra were then searched using MS Biotools (version 3.2) program against the taxonomy Viridiplantae (Green plants).

### In situ localization

The full-length coding sequence of RiD was cloned into pENTR D-TOPO vector (Invitrogen, CA, USA) using RiDloc-F and RiDloc-R (Additional file 4: Table S1) according to the manufacturer’s instructions. And then it was cloned into destination vector pEARLY GATE 101 (CD3-683, YFP) using LR clonase (Invitrogen, CA, USA). YFP was chosen over GFP due to its higher photo-stability and improved brightness (Shaner et al. 2005). The destination clone was finally coated with gold particles (Bio-Rad, CA, USA) and was bombarded into onion epithelial cells using PDS-1000/He System (Bio-Rad, CA, USA). The cells were observed after 24 h in Leica Zeiss Confocal Laser scanning microscope at 488 nm excitation and fluorescence emission signal recovery between 505 and 535 nm.

### Artificial diet based bioassay of *L. erysimi* with purified RiD and BjD

Second instar nymphs were used for this experiment. Artificial diet was formulated using the original description by Dadd and Mittler (1976). The experiment was set up in small 35 mm petri dishes (Tarsons, Kolkata, India) with small perforated bottom just to allow the passage of air. Twenty nymphs of *L. erysimi* were incubated for each set and the petri dishes were covered with parafilm stretched 4 times to its original size. A liquid diet of 400 µL supplemented with different doses of RiD (0, 5, 10, 15, 20 and 25 µg/mL each) was placed on each parafilm and was covered with another parafilm to make a pouch. The entire experiment was done in triplicate. Another experiment was set up with a range of different doses of BjD (0, 10, 20, 30, 40 and 50 µg/mL each). Water was used as a no diet control to show that the provided liquid diet (without RiD and BjD) in comparison with water, can support the survival of the aphids. Aphid survivability was then recorded at every 12 h interval. Abbot’s formula was used to calculate the corrected mortality (Abbott 1925). Statistical Probit analysis using the χ^2^ method (Chi 1997) was performed to calculate the LC_50_ value of RiD and BjD against *L. erysimi*. Statistical significance level used was 0.05 (α ≤ 0.05).

## Additional files

10.1186/s40064-016-2144-2 Representative images of *R. indica* and *B. juncea* infested with *L. erysimi*.
10.1186/s40064-016-2144-2 MALDI-TOF/TOF–MS spectra of peptides generated from tryptically digested RiD.
10.1186/s40064-016-2144-2 SignalP-NN Prediction plot, and representation of the RiD: YFP fusion protein.
10.1186/s40064-016-2144-2 Sequence of the all the primers used.

## Abbreviations

BjD: *Brassica juncea* defensin
dpi: day(s) post-infestation
ER: endoplasmic reticulum
GFP: green fluorescent protein
hpi: hour(s) post-infestation
HCCA: α-cyano-4-hydroxycinnamic acid
IPTG: isopropyl β-d-1-thiogalactopyranoside
LC_50_: lethal concentration which causes 50 % mortality of test population
RiD: *Rorippa indica* defensin
MALDI-TOF/TOF: matrix-assisted laser desorption/ionization time-of-flight
Ni–NTA: nickle–nitrilotriacetic acid
PAGE: polyacrylamide gel electrophoresis
SDS: sodium dodecyl sulfate
TFA: trifluoroacetic acid
YFP: yellow fluorescent protein
