# Supplementary material for: Functional characterization of Rorippa indica defensin and its efficacy against Lipaphis erysimi
Source: Springerplus. 2016 Apr 23;5:511. doi: 10.1186/s40064-016-2144-2 (PMC4842206; doi:10.1186/s40064-016-2144-2)
Supplement: Supplementary file 2 — 10.1186/s40064-016-2144-2 MALDI-TOF/TOF–MS spectra of peptides generated from tryptically digested RiD. [file 40064_2016_2144_MOESM2_ESM.docx]

Figure S2, Supplementary Material for

Title: **Functional characterization of *Rorippa indica* defensin and its efficacy against *Lipaphis erysimi***

Authors: Poulami Sarkar^1^, Jagannath Jana^2^, Subhrangshu Chatterjee^2^ and Samir Ranjan Sikdar^1^

^1^Division of Plant Biology, Centenary Campus, Bose Institute, Kolkata-700054, India

^2^Department of Biophysics, Centenary Campus, Bose Institute, Kolkata-700054, India

Address for correspondence: [samir@jcbose.ac.in](mailto:samir@jcbose.ac.in). Fax:     +91-33-2355-3886


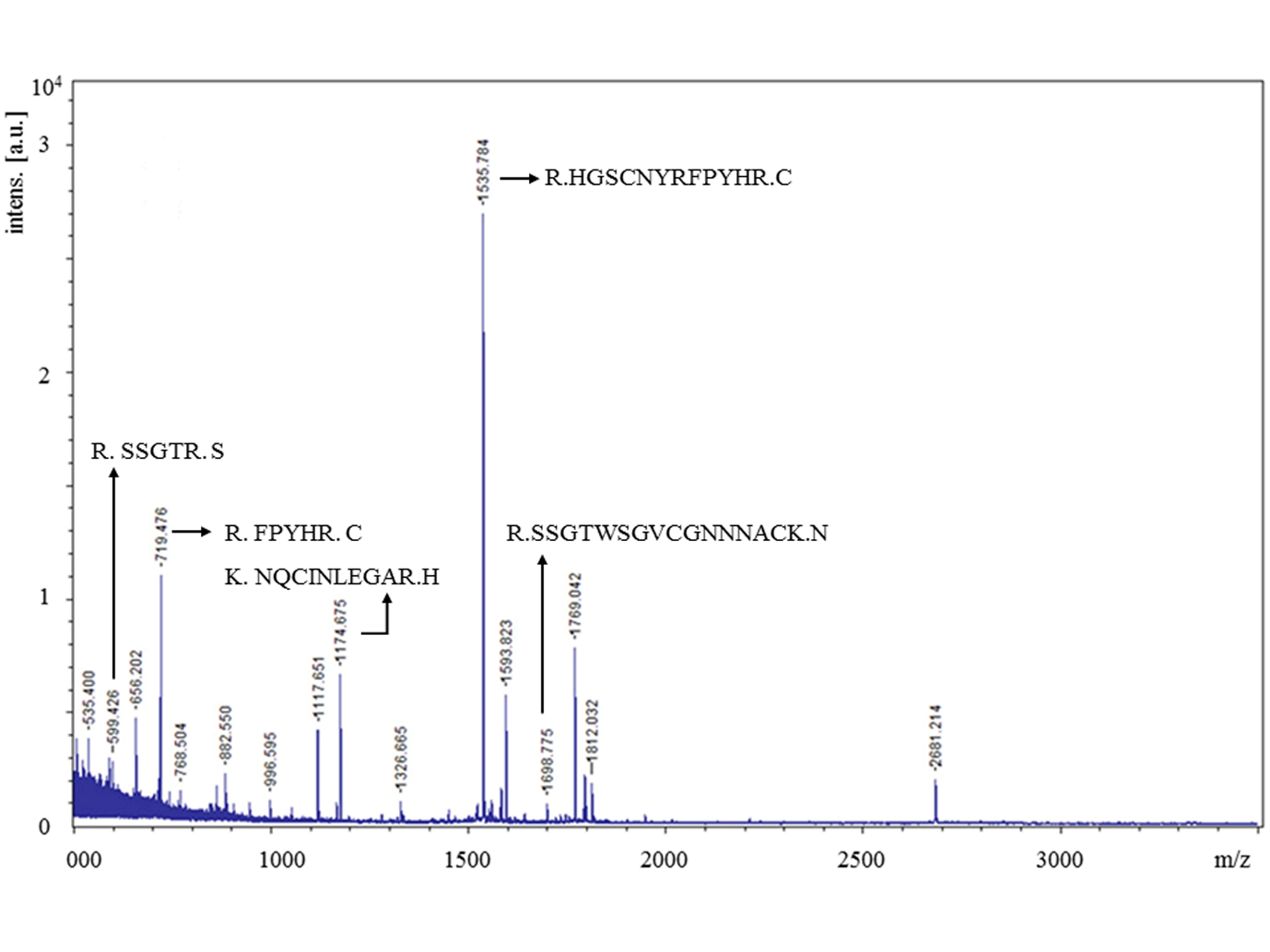


Supplementary Fig. 2. MALDI-TOF/TOF-MS spectra of peptides generated from tryptically digested RiD.
