# Supplementary material for: Functional characterization of Rorippa indica defensin and its efficacy against Lipaphis erysimi
Source: Springerplus. 2016 Apr 23;5:511. doi: 10.1186/s40064-016-2144-2 (PMC4842206; doi:10.1186/s40064-016-2144-2)
Supplement: Supplementary file 3 — 10.1186/s40064-016-2144-2 SignalP-NN Prediction plot, and representation of the RiD: YFP fusion protein. [file 40064_2016_2144_MOESM3_ESM.docx]

Figure S3, Supplementary Material for

Title: **Functional characterization of *Rorippa indica* defensin and its efficacy against *Lipaphis erysimi***

Authors: Poulami Sarkar^1^, Jagannath Jana^2^, Subhrangshu Chatterjee^2^ and Samir Ranjan Sikdar^1^

^1^Division of Plant Biology, Centenary Campus, Bose Institute, Kolkata-700054, India

^2^Department of Biophysics, Centenary Campus, Bose Institute, Kolkata-700054, India

Address for correspondence: [samir@jcbose.ac.in](mailto:samir@jcbose.ac.in). Fax:     +91-33-2355-3886


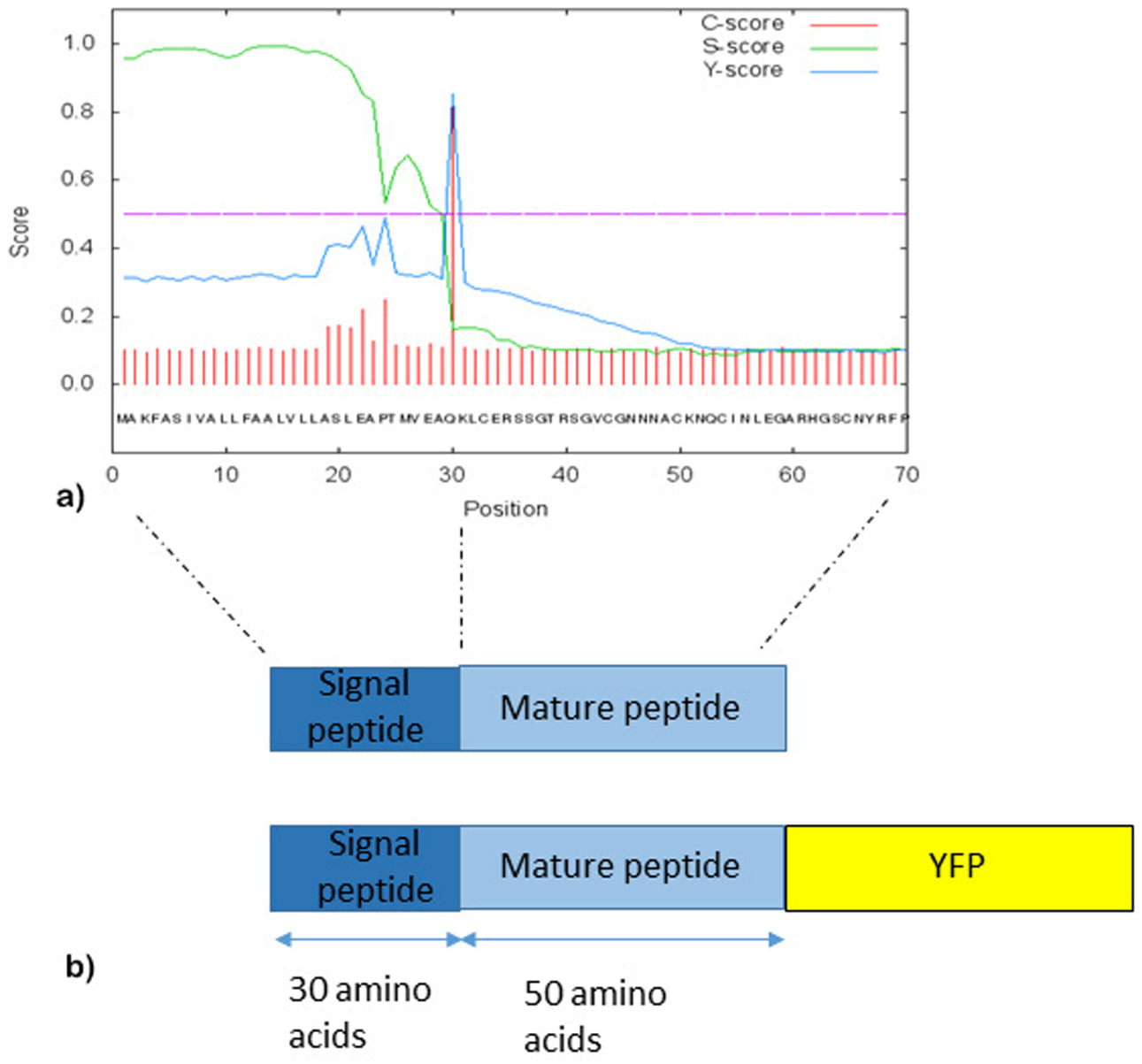


Supplementary Fig. 3. a) SignalP-NN Prediction plot (http://www.cbs.dtu.dk/services/signalP) of the RiD protein sequence. The graph shows the cleavage site score (C-score) and the signal peptide score (S-score). The Y-score (i.e. the combination of C-score and S-score) predicts the presence of an N-terminal signal peptide with a cleavage site between position 30 and 31. (C score, red plot; S score, green plot; Y score, blue plot). b) Representation of the RiD protein; the RiD::YFP fusion protein used for stable and transient expressions in onion cell.
