# Supplementary material for: Functional characterization of Rorippa indica defensin and its efficacy against Lipaphis erysimi
Source: Springerplus. 2016 Apr 23;5:511. doi: 10.1186/s40064-016-2144-2 (PMC4842206; doi:10.1186/s40064-016-2144-2)
Supplement: Supplementary file 4 — 10.1186/s40064-016-2144-2 Sequence of the all the primers used. [file 40064_2016_2144_MOESM4_ESM.docx]

Table S1, Supplementary Material for

Title: **Functional characterization of *Rorippa indica* defensin and its efficacy against *Lipaphis erysimi***

Authors: Poulami Sarkar^1^, Jagannath Jana^2^, Subhrangshu Chatterjee^2^ and Samir Ranjan Sikdar^1^

^1^Division of Plant Biology, Centenary Campus, Bose Institute, Kolkata-700054, India

^2^Department of Biophysics, Centenary Campus, Bose Institute, Kolkata-700054, India

Address for correspondence: [samir@jcbose.ac.in](mailto:samir@jcbose.ac.in). Fax:     +91-33-2355-3886

Supplementary Table 1: Sequence of the all the primers used.

| **S. No.** | **Primer name** | **Sequence of the primers (5′-3′)** |
| --- | --- | --- |
|  | **Primers used for 3′ RACE** | |
| **1.** | 3PDF1 | TCAACGCAGAGTACATGGGG |
| **2.** | 3PDF2 | GTGCGAGAGGTCAAGTGGAA |
|  | **Primers used for 5′ RACE** | |
| **3.** | 5PDF1 | ACACACATGTCATAAAGTCGCT |
| **4.** | 5PDF2 | ACACACATGTCATAAAGTCGCT |
|  | **Primers used for 3′ Genome Walk** | |
| **5.** | 3-GSP1 | CCCATGTTAATCTACCCAATAAGCT |
| **6.** | 3-GSP2 | CGCTCTATGAGCGACTTTATGACATGTGT |
|  | **Primers used for 5′ Genome Walk** | |
| **7.** | 5-GSP1 | GCCATGACTATTAATTACTACTTTGTTTT |
| **8.** | 5-GSP2 | CCCATGTACTCTGCGTTGATACCACTGTT |
|  | **Primers to clone the full length sequence of RiD** | |
| **9.** | RiDForward | ATGGCTAAGTTTGCTTCCATCGT |
| **10.** | RiDReverse | TTAACATGGGAAGTAACAGATACAT |
|  | **Primers used for protein expression of RiD** | |
| **11.** | RiDexp-F | TTTTGGATCCAAGTTGTGCGAGA |
| **12.** | RiDexp-R | AAAAGAGCTCTTAACATGGGAAGTAAC |
|  | **Primers used for protein expression of BjD** | |
| **13.** | BjDexp-F | TTTTGGATCCAAGTTGTGCCAGA |
| **14.** | BjDexp-R | AAAAGAGCTCTTAACAAGGGAAGTAGCA |
|  | **Primers used for Localization studies** | |
| **15.** | RiDloc-F | CACCATGGCTAAGTTTGCTTCCAT |
| **16.** | RiDloc-R | ACATGGGAAGTAACAGATACATCGG |

GSP- Gene specific primer

PDF1- Plant defensin first primer

PDF2- Plant defensin nesting primer

RiD- *Rorippa indica* defensin

BjD- *Brassica juncea* defensin
